# Long term efficacy and safety of canakinumab in children with systemic juvenile idiopathic arthritis with and without fever

**DOI:** 10.1186/1546-0096-13-S1-O83

**Published:** 2015-09-28

**Authors:** G Horneff, N Ruperto, H Brunner, P Quartier, T Constantin, E Alexeeva, I Kone-Paut, K Marzan, N Wulffraat, R Schneider, S Padeh, V Chasnyk, C Wouters, J Kummerle Deschner, T Kallinich, B Lauwerys, E Haddad, E Nasonov, M Trachana, O Vougiouka, K Abrams, K Leon, K Lheritier, A Martini, D Lovell

**Affiliations:** 1 PRINTO-Istituto Gaslini, Genova, Italy; 2 PRCSG, Cincinnati, OH, United States; 3 Necker-Enfant Malades Hospital, Paris, France; 4 Cliniques Universitaires Saint-Luc and Université Catholique de Louvain, Brussels, Belgium; 5 Novartis Pharmaceuticals Corporation, East Hanover, NJ, USA; 6 Novartis Pharma AG, Basel, Switzerland

## Introduction

Rapid and sustained efficacy of canakinumab (CAN) were previously demonstrated in patients with systemic juvenile idiopathic arthritis (SJIA) [1]. However, little is known about potential differences in response to CAN treatment between patients with vs. without SJIA-associated fever at the time of the first CAN administration.

## Objectives

To evaluate the long-term efficacy and safety profile of CAN-naïve SJIA patients with and without SJIA-associated fever.

## Patients and methods

patients aged 2-20 years with SJIA with and without SJIA associated fever at enrollment received open-label CAN 4mg/kg s.c. every 4 wks. Every 3 months, response to CAN was measured by adapted JIA ACR response criteria (aACR/JIA); juvenile arthritis disease activity score (JADAS); clinical inactive disease; clinical remission on medication (CRM, 6 months continuous clinical inactive disease). Safety was assessed monthly.

## Results

Data on 122/267 patients, 53 (43%) with and 69 (57%) without SJIA associated fever, were available for analysis with a median 94 wk study duration. At Wk4, ~75% of both subgroups had responded (≥ aACR/JIA30), increasing to 90% at Wk12. At Wk2, ~21% of both subgroups had inactive disease; 44% at Wk8; 60% at Wk20 and then 60-70% for the remainder of the trial. CRM was achieved in about 29% of patients in both subgroups with ~22% maintaining it for ≥12 consecutive months. At baseline, the median JADAS score was 21.5 with 8 (7.5%) and 99 (92.5%) patients meeting the criteria for moderate (JADAS >3.8 and ≤10.5) and high disease activity (JADAS >10.5), respectively. At Day 15, the median JADAS was 6.8 and 1.5 at the last assessment. At the last assessment, 53 (48%) patients had inactive disease (JADAS ≤ 1); 10 (9%) with low active disease activity (JADAS >1 and ≤3.8); while 14 (13%) had moderate and 31 (28%) with high disease activity. Infection (0.56 infections/100 patient-days), typically involving upper respiratory tract was the most common type of adverse event. Fifteen patients discontinued due to an AE and 40 had >1 SAE (mostly infections, macrophage activation syndrome (MAS), or flare-associated) and no deaths. Eight cases of MAS (0.013 events/100 patient-days) were reported.

## Conclusion

Canakinumab provides similar efficacy in SJIA patients with and without SJIA-associated fever at treatment onset. The long-term safety profile was acceptable and similar to the pivotal program in SJIA children with fever at enrollment.
